# Localized Tacrolimus Delivery for Peripheral Nerve Regeneration: Molecular Mechanisms, Biomaterial Platforms, and Translational Strategies

**DOI:** 10.3390/ijms27104179

**Published:** 2026-05-08

**Authors:** Ramkumar Katturajan, Sara N. Shah, Jordan Crabtree, Arif Hussain, Konstantin Feinberg, J. Paul Santerre, Gregory H. Borschel

**Affiliations:** 1 Division of Plastic Surgery, Indiana University, Indianapolis, IN 46202, USA; rkattura@iu.edu (R.K.);; 2 Institute of Biomedical Engineering, University of Toronto, Toronto, ON M5G 1M1, Canada; 3 Faculty of Dentistry, University of Toronto, Toronto, ON M5G 1M1, Canada

**Keywords:** tacrolimus, peripheral nerve injury, local drug delivery, biomaterials, neuroregeneration, translational medicine, FK506, nerve repair, polymer

## Abstract

Peripheral nerve injuries cause profound medical and socioeconomic consequences. Despite substantial microsurgical advances, including nerve autografting, nerve transfers, and the commercial availability of effective conduits, functional recovery remains incomplete for most patients. Current outcomes underscore the need for novel adjunctive therapies capable of enhancing axonal regeneration, accelerating reinnervation, and mitigating denervation-induced target atrophy. Tacrolimus, a calcineurin inhibitor widely used in organ transplantation, has emerged as a potent immunomodulatory and neuroregenerative agent. However, its systemic use is constrained by severe dose-limiting toxicities and metabolic derangements. This limitation has driven a paradigm shift toward localized tacrolimus delivery, leveraging biomaterials to achieve therapeutic drug concentrations at the repair site while minimizing systemic toxicity. This review synthesizes the state-of-the-art advances in biomaterial-based tacrolimus local delivery systems. We highlight biological mechanisms underlying tacrolimus-mediated neuroregeneration and immunomodulation. Engineering strategies including nerve conduits, wraps, injectable hydrogels, electrospun scaffolds, and stimuli-responsive carriers are discussed, with attention to polymeric composition, fabrication technologies, degradation kinetics, and pharmacological performance. We also explored the regulatory, manufacturing, and scalability challenges inherent to drug–device combination products. Finally, we identify emerging directions including multimodal biomaterials that integrate tacrolimus with trophic factors, extracellular vesicles, or bioelectrical stimulation. Collectively, biomaterial-enabled tacrolimus delivery represents a transformative strategy to bridge traditional nerve surgical repair and functional recovery. This review provides a roadmap for future interdisciplinary innovation at the interface of biomaterials science, neurobiology, pharmacology, and surgery.

## 1. Introduction

Peripheral nerve injuries (PNIs) affect millions of people globally each year, often leading to debilitating long-term functional deficits, chronic pain, and substantial socioeconomic burden [1,2,3]. These injuries commonly arise from disease or trauma, and sensory loss, motor paralysis, and neuropathic pain severely impact quality of life [3,4]. In the United States alone, approximately 20 million individuals are affected annually, with traumatic nerve injuries representing nearly 5% of emergency room visits [5,6]. Lesions exceeding 3 cm in length or injuries treated after substantial delay are associated with poorer outcomes [6,7]. While the peripheral nervous system (PNS) possesses regenerative potential, 2–5% of patients develop chronic motor and sensory deficits and persistent neuropathic pain, often compounded by secondary complications such as joint stiffness [5,8,9]. Both the diagnosis and management of PNIs are complicated by differential injury mechanisms, comparatively poor regenerative capacity, and the limited timeframe in which interventions can achieve demonstrable recovery. While minor PNIs may recover spontaneously, severe cases including crush injuries often result in extensive axonal loss or long-gap defects requiring surgical intervention. This may include direct nerve repair or nerve grafting, although complete functional recovery remains elusive despite advances in these surgical techniques [10,11]. The complex post-injury environment is characterized by Wallerian degeneration, inflammation, glial scarring, and denervated target muscle atrophy. Together, these factors create formidable barriers to successful axonal regeneration and functional reinnervation, highlighting the urgent need in novel therapeutic strategies capable of enhancing and expediting nerve regeneration [4]. Current nerve repair methods have not advanced appreciably in several decades, with contemporary surgeons relying on mechanically joining nerve ends during microsurgical nerve coaptation. In addition, no adjunctive pharmaceutical agents have been approved by the FDA or other regulatory agencies to enhance the outcomes of nerve repair. Given the persistent limitations in nerve regeneration, and the explosion in biomaterials research over the last decade, the field is ripe for disruptive innovations.

Tacrolimus (FK506), a potent calcineurin inhibitor widely recognized for its immunosuppressive properties in organ transplantation, has emerged as a promising neuroregenerative agent. Discovered in 1987 and approved for clinical use in the early 1990s, tacrolimus later demonstrated direct neurotrophic properties promoting axonal outgrowth, neuronal survival, and myelination beyond its immunomodulatory effects [12,13,14]. This dual functionality positions it as an attractive candidate for improving outcomes in nerve repair [14]. However, systemic administration of tacrolimus is severely limited by its tight safety margin and a spectrum of adverse side effects, including multiorgan toxicity and non-targeted immunosuppression. These toxicities often necessitate dose reductions or treatment discontinuation, hindering the full realization of its neuroregenerative potential in PNIs [13,15,16].

The inherent limitation of systemic tacrolimus administration has motivated the development of localized delivery strategies (LDSs), which concentrate the drug at the site of injury to maximize its efficacy while minimizing systemic exposure and adverse effects [17]. This targeted approach improves the overall safety profile of the drug and may potentially enable a more prolonged and effective treatment. While prior reviews have summarized the general neuroregenerative potential of tacrolimus, the present review specifically focuses on localized biomaterial-enabled delivery systems, release engineering, regulatory considerations, and translational device design relevant to clinical nerve repair.

## 2. Biological Barriers to Functional Recovery After Nerve Injury

Surgical reconstruction remains the primary therapeutic approach for managing PNIs characterized by extensive axonal disruption [18]. Common surgical techniques include nerve autografting, nerve allografting, nerve transfers, and biomaterial-based interventions involving nerve conduits and hydrogels [19,20]. Among these approaches, nerve autografting is considered the gold standard due to the favorable biological properties of native tissue including the presence of Schwann cells, neurotrophic factors, and basal lamina that promote axonal regeneration [21]. However, autografts are constrained by donor site morbidity, finite tissue availability, and diminished efficacy in nerve gaps exceeding 6 cm [11,22]. Fresh (cellular) nerve allografts provide an abundant source of transplant material for large nerve defects; however, their clinical utility has been historically limited by immune rejection mediated through direct and indirect allo-recognition pathways, necessitating systemic immunosuppressive therapy. In contrast, acellular nerve allografts derived from decellularized cadaveric tissue exhibit reduced immunogenicity and have gained clinical adoption, although their efficacy remains limited in long-gap and mixed motor–sensory nerve reconstruction [23]. Nerve transfers provide an advantage by leveraging donor nerves near the neuromuscular junction, but their clinical utility is restricted by surgical complexity and the requirement for healthy donor nerves [22]. Meanwhile, biomaterials including nerve conduits and hydrogels have shown promise for small defects, but are less effective in bridging gaps greater than 2–3 cm [24]. Despite advances in microsurgical techniques, complete functional recovery remains exceptionally rare, with fewer than 55% of patients achieving satisfactory motor or sensory outcomes [7,25]. The discrepancy between anatomical repair and functional restoration is largely attributed to persistent biological barriers, including Wallerian degeneration, chronic inflammation, fibrosis, and prolonged denervation [26,27,28].

### 2.1. Time-Dependent Denervation Effects on Musculature and Nerves

The viability of both denervated muscle and the nerves that supply them declines in a time-dependent manner. With prolonged denervation, muscle fibers undergo marked cellular alterations, including ionic imbalances, decreased resting membrane potential, and accelerated protein breakdown. These changes culminate in progressive degradation of the muscle’s contractile apparatus and reduced contractility [29]. Chronically denervated muscles exhibit fiber loss, atrophy, and fibrotic remodeling characterized by dense collagen deposition and adipocyte infiltration [30]. This process is termed chronic axotomy, and it occurs when neurons in the proximal stump are no longer connected to their target end-organs, triggering a downstream effect in the distal stump where Schwann cells are disconnected from neuronal cell bodies and any viable axons [31]. Consequently, the distal environment becomes progressively less receptive to regenerating axons.

### 2.2. Axonal Regeneration Rate Limitations

Another major barrier to successful nerve regeneration and functional restoration is the naturally slow rate of axonal regeneration. Axons typically regenerate at approximately 1 mm per day, likely constrained by the rate of Schwann cell extension rather than intrinsic axonal growth [24]. Over long distances, this limited rate allows for degeneration of the target muscles and distal nerve pathways before reinnervation occurs. As discussed in Section 2.1, prolonged denervation compromises the distal target environment and further diminishes the likelihood of successful reinnervation. Accordingly, these rate-based limitations highlight the importance of innovative therapeutic strategies to accelerate axonal elongation and improve early regeneration.

### 2.3. Distal Pathway Degeneration

Following nerve injury, the distal nerve segments undergo Wallerian degeneration, a tightly regulated process and hallmark consequence of peripheral nerve damage [31]. Initiated within 3–4 days, Wallerian degeneration is characterized by the loss of cell membrane integrity and granular disintegration of the axonal cytoskeleton due to disconnection of the cell bodies from their metabolic sources. The cessation of retrograde and anterograde transport leads to swelling at both ends of the damaged neuron [32]. Subsequently, due to the absence of axonal connections, Schwann cells dedifferentiate, demyelinate, and activate resident macrophages. The cellular coordination between Schwann cells and infiltrating immune cells initiates the phagocytosis of myelin debris, effectively priming the distal nerve site for regeneration [33]. As a result, the dedifferentiated Schwann cells proliferate along the residual extracellular matrix (ECM), forming the bands of Büngner, which create a path for the regenerating axon to regrow [32]. With prolonged denervation, however, these supportive distal pathways gradually become less conducive to regrowth. Schwann cells atrophy and the endoneurial tubes, which serve as guiding structures, become progressively occluded by collagen filaments. These structural changes severely limit motoneuron axonal regeneration, potentially reducing successful reinnervation to less than 10% in chronic neurodegenerative conditions [12].

### 2.4. Inflammation and Scar Tissue Formation

Inflammation and scar tissue formation hinder nerve regeneration by creating a hostile biochemical environment and promoting misdirected axonal elongation. An acute inflammatory response is initiated by neutrophils and activated resident macrophages, which secrete pro-inflammatory cytokines such as tumor necrosis factor (TNF)-α, interleukin (IL)-6, chemokines (CXCL8 and CCL2), and IL-12 [34,35]. Timely resolution of inflammation is essential to support early regeneration. If the inflammatory agents persist, this acute response can progress to chronic inflammation, as fibroblast activation drives the formation of fibrotic scar tissue at the distal nerve stump [25]. This post-injury fibrosis results in the excessive accumulation of stiff collagen fibers and ECM proteins, increasing tissue rigidity and impeding axonal growth toward the distal nerve [36]. Following PNI, regenerated motor axons can produce lateral buds that innervate random Schwann cells rather than their appropriate targets [37]. Over time, this misdirection results in aberrant motor or sensory innervation due to crossed signaling from motor axons targeting inappropriate sensory end organs [37].

A clinical example is neurotrophic keratopathy (NK), where injury to the trigeminal nerve pathway leads to the loss of corneal innervation. Consequently, the loss of nerve-derived trophic support leads to epithelial breakdown, impaired wound healing, and progressive vision loss. Persistent epithelial defects perpetuate chronic inflammation, stromal scarring, and fibrosis, further exacerbating the hostile environment for nerve regeneration and epithelial repair [38,39]. Clinically, this manifests as reduced functional specificity, as regenerated axons often fail to re-establish connections with their appropriate targets, thereby diminishing sensory recovery and corneal integrity [2] as NK progresses to permanent blindness.

### 2.5. Immunological Challenges

Fresh nerve allografting is a surgical option for bridging large nerve gaps, providing a rich ECM and growth factors that support regeneration. Allogeneic grafts, or allografts, are tissues that are transferred between two genetically disparate individuals of the same species [22,40]. They are favorable for large segmental PNIs and are advantageous due to their accessibility and ready supply. However, their clinical use is constrained by the risk of immune rejection. Host T-cell and macrophage responses target donor Schwann cells and extracellular components, leading to graft degeneration and fibrotic replacement. This rejection occurs through both direct and indirect allo-recognition [23]. In direct allo-recognition, the recipient’s T-cells are activated by donor dendritic cells (DCs) presenting allogeneic HLA molecules. For indirect allorecognition, the alloreactive T-cells are stimulated to react against the transplant by recognizing processed subcellular material presented by the recipient’s own DCs [41]. Preventing rejection typically requires systemic immunosuppression, which introduces substantial risks and limits the long-term applicability of allografting.

## 3. Mechanism of Action of Tacrolimus

Understanding the mechanisms by which tacrolimus influences immune regulation and nerve regeneration is critical for translating its experimental benefits into clinical strategies for peripheral nerve repair. Beyond its established use in transplantation medicine, tacrolimus modulates inflammatory responses and enhances axonal regeneration, creating a biological environment conducive to functional recovery after nerve injury. This section summarizes the key immunological and neuroregenerative pathways through which tacrolimus exerts its therapeutic effects.

### 3.1. Immunomodulation

**Figure 1:**
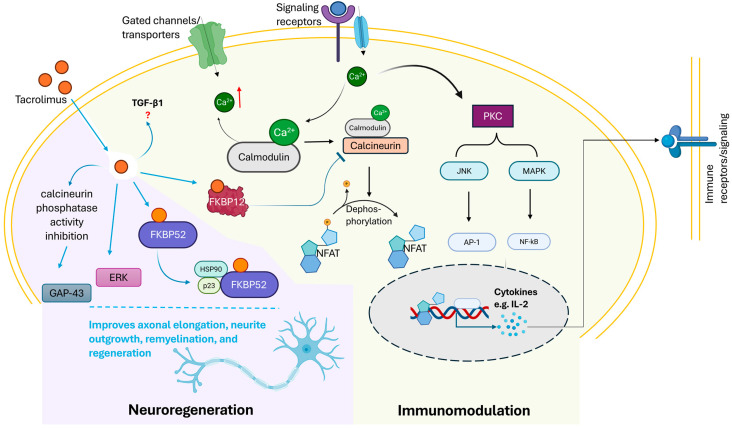
Immunomodulatory and neuroregenerative mechanisms of tacrolimus. Tacrolimus binds FKBP12, inhibiting the calcium/calmodulin-dependent phosphatase calcineurin. This blocks NFAT nuclear translocation, reducing IL-2 production and T-cell activation. Concurrent modulation of JNK, MAPK, and ERK signaling, along with the potential upregulation of TGFβ1, collectively suppresses neuroinflammation, limits tissue damage, and promotes a regenerative environment that supports axonal growth and graft survival. Created in BioRender. Katturajan, R. (2026) https://BioRender.com/shaakmn, accessed on 27 April 2026.

Tacrolimus, also referred to as FK506, is a potent immunosuppressive agent widely utilized in organ transplantation that has also demonstrated benefits in peripheral nerve repair through neurotrophic mechanisms distinct from immune suppression, with immunomodulatory effects suggested to contribute indirectly [13,42]. Mechanistically, tacrolimus functions as a calcineurin inhibitor by forming a complex with the intracellular immunophilin, FK506-binding protein 12 (FKBP12), which binds to and inhibits calcineurin, a calcium/calmodulin-dependent phosphatase. This blockade prevents the dephosphorylation and nuclear translocation of the nuclear factor of activated T-cells (NFAT), thus suppressing the transcription of key pro-inflammatory cytokines such as IL-2 [43,44,45]. By suppressing IL-2 production, tacrolimus markedly inhibits T-cell activation and proliferation, thereby dampening the adaptive immune response central to graft rejection [46] and neuroinflammation in nerve injury [47] (Figure 1).

Additionally, tacrolimus modulates other immune signaling pathways, notably the c-Jun N-terminal kinase (JNK) and p38 mitogen-activated protein kinase (MAPK) cascades, further attenuating T-cell activation and reducing the expression of inflammatory cytokines important for early immune responses and tissue injury [48]. In support of this immune signaling modulation, tacrolimus has been shown to attenuate fibrotic scar formation following peripheral nerve injury. For instance, in a rat sciatic nerve injury model, FK506 significantly reduced the perineural scar area and fibroblast density at the nerve repair site, an effect attributed to the inhibition of fibroblast proliferation and induction of apoptosis via JNK-associated pathways [49]. This multi-pathway influence also moderately inhibits B-cell function and natural killer (NK) cell activity, further broadening the immunomodulatory scope of tacrolimus and contributing to a regenerative microenvironment permissive for axonal growth. A proposed mechanism, though not fully elucidated, also involves the modulation of transforming growth factor-beta 1 (TGF-β1), which may provide additional immunoregulatory control but has also been linked to fibrotic side effects such as nephrotoxicity [50]. However, in the context of nerve injury, this targeted suppression of immune activation, inflammatory cytokine milieu, and scar formation is crucial. It fosters a pro-regenerative environment, limits secondary tissue damage, and supports graft survival and axonal regeneration by mitigating destructive immune-mediated processes [51]. Thus, through the targeted inhibition of calcineurin and related signaling pathways, tacrolimus orchestrates immunosuppression and immune modulation that is favorable for peripheral nerve repair and regeneration following injury.

### 3.2. Neuroregeneration

In a serendipitous discovery over 30 years ago, Gold and colleagues observed that tacrolimus increases the rate of axon regeneration in preclinical models [52]. Beyond its immunosuppressive roles, tacrolimus exerts distinct neuroregenerative effects through well-characterized mechanisms, with extensive evidence supporting its efficacy in mitigating denervation atrophy [53], prolonged axotomy [54], distal pathway denervation, and promoting axonal and neurite growth [55]. Through its molecular interactions, tacrolimus binds to FK506-binding proteins, particularly FKBP52, which forms complexes with heat shock protein 90 (Hsp90) and co-chaperone p23 within the neuronal nuclei [56]. This complex subsequently redistributes to the growth cones of regenerating neurites, guiding their extension in response to chemotactic signals and accelerating axonal elongation [57]. As a result, tacrolimus promotes faster axonal regeneration, increased axon diameter and myelination, and earlier recovery of motor function in nerve injury models [58].

Tacrolimus further enhances neurite outgrowth by activating signaling pathways such as the extracellular signal-regulated kinase (ERK) cascade, mediated through p23, which is critical for neurite elongation [59,60]. By inhibiting calcineurin phosphatase activity, tacrolimus increases the phosphorylation of growth-associated protein 43 (GAP-43) [61], a critical regulator of growth cone formation and axonal pathfinding [62]. Site-specific phosphorylation of GAP-43 induced by tacrolimus sustains or reinduces its expression, fostering axonal branching and regenerative capacity [63] (Figure 1).

In the context of denervation atrophy and distal nerve pathway degeneration, tacrolimus supports muscle preservation and remyelination by accelerating axonal regrowth while maintaining neurotrophic support. It promotes Schwann cell proliferation and modulates gene expression to enhance neurotrophin secretion, bolstering the repair microenvironment essential for functional recovery following prolonged injury or delayed nerve repair [33,64]. Collectively, the pro-neuroregenerative effect of tacrolimus is mediated on three levels: (i) protecting and inducing axonal growth, (ii) supporting axo-glial interaction, and consequently, (iii) protecting and supporting distal target tissues.

## 4. Systemic Tacrolimus: Limitations and Risks

Tacrolimus is classified as a Biopharmaceutics Classification System (BCS) class II drug, characterized by poor water solubility, yet high permeability. It exhibits a narrow therapeutic index, typically requiring blood concentrations between 5–20 ng/mL to optimize efficacy and minimize toxicity [65]. With an approximate half-life of 12 h [66] and highly variable oral bioavailability, tacrolimus often requires individualized dosing and meticulous therapeutic drug monitoring [67]. Fluctuations in systemic drug levels can lead to under-immunosuppression and graft rejection, or conversely, to toxicity with systemic adverse events [68].

The adverse effects associated with the systemic administration of tacrolimus can be severe and life-threatening. Nephrotoxicity and the potential to cause acute or chronic kidney damage requires vigilant dose monitoring, particularly in patients with pre-existing renal impairments [13]. Neurotoxicity is also frequently observed, ranging from tremors and headaches to more severe manifestations such as seizure or vision loss due to optic nerve damage [69,70]. Furthermore, metabolic complications may occur, including hyperglycemia, post-transplant diabetes, hypertension, and hyperuricemia. Additional adverse effects include gastrointestinal disturbances, increased susceptibility to infections, and alterations in hair growth, including alopecia or hypertrichosis [71].

Notably, systemic adverse effects have also been reported following topical administration, particularly at elevated concentrations or with prolonged use, indicating potential systemic absorption even from local applications [72]. Patient-specific factors such as age, comorbidities, and concurrent medications can further complicate overall health management due to the extensive metabolism of tacrolimus by CYP3A enzymes [73]. Collectively, these limitations highlight the need for safer delivery systems that preserve the therapeutic benefits of tacrolimus while minimizing systemic exposure. Advanced drug delivery platforms such as implants, nanocarriers, and targeted formulations offer promising strategies to overcome these limitations.

## 5. Local Delivery System for Tacrolimus in Nerve Repair

Local delivery systems (LDSs) are engineered to enhance the targeted delivery, bioavailability, and sustained release of therapeutic agents. By concentrating drugs at the site of injury, LDSs can reduce systemic toxicity and enable lower dosing without compromising therapeutic efficacy. This is especially important for tacrolimus due to its breadth of systemic side effects [74]. Moreover, sustained release maintains consistent therapeutic levels that may improve patient compliance. Although systemic tacrolimus facilitates neuroregeneration [75], systemic administration is limited by the side effects described above [76]. Therefore, LDSs that achieve high therapeutic concentrations while minimizing systemic exposure are highly desirable for tacrolimus. The following section describes various biomaterial-based delivery systems specifically designed for nerve injury applications. These platforms leverage advanced tissue engineering principles to provide targeted drug delivery as well as structural and biochemical support for nerve regeneration.

### 5.1. Nerve Guidance Conduits

Nerve guidance conduits (NGCs) are fundamental to modern peripheral nerve repair, providing a physical bridge across nerve gaps and directional guidance for regenerating axons. When incorporated with therapeutic agents, NGCs function as dual-purpose devices, delivering sustained local therapy while maintaining structural guidance for axonal growth [77,78,79]. The design of these conduits, from their material composition to their internal architecture, is critical for modulating release and promoting a favorable microenvironment for neuroregeneration.

Several biomaterials have been investigated for delivery in nerve regeneration, each offering distinct benefits. Natural polymers such as collagen, fibrin, silk fibroin, chitosan, and decellularized nerve matrices are highly biocompatible and mimic the ECM, promoting cell adhesion and neurite outgrowth [80]. In contrast, synthetic polymers including polylactic acid (PLA), polyglycolic acid (PGA), polycaprolactone (PCL), and especially poly(lactic-co-glycolic acid) (PLGA) provide tunable degradation kinetics, mechanical strength, and sustained localized drug release, rendering them ideal for controlled therapeutic delivery [81]. Hybrid or composite biomaterials combine the benefits of both natural and synthetic polymers, often pairing structural integrity with enhanced biocompatibility [82]. Moreover, the incorporation of conductive elements such as graphene or carbon nanotubes is an emerging strategy being studied for the introduction of bioelectrical functionality, further supporting nerve repair [83].

These polymers are synthesized by fabrication technologies that critically influence the architecture and drug delivery capabilities of drug-loaded NGCs. Electrospinning facilitates the formation of aligned nanofibers that closely mimic native nerve structures, simultaneously guiding axonal growth and providing sustained drug release [19]. Three-dimensional (3D) printing offers high precision in fabricating complex conduit designs, including multichannel lumens and biomimetic patterns, while permitting the integration of drug reservoirs for controlled release [84]. Additionally, conventional fabrication methods such as solution casting, freeze-drying, and salt leaching are widely used to produce porous scaffolds that support cell infiltration, nutrient diffusion, and localized drug release [85]. Drug incorporation methods critically influence release, particularly for tacrolimus. Various drug-loading strategies have been established to incorporate therapeutics into NGCs, each yielding distinct release profiles. Direct incorporation involves dissolving or suspending tacrolimus within the polymer solution prior to conduit formation, enabling sustained drug release concurrent with material degradation. Surface modification or coating deposits tacrolimus onto the exterior layer of the conduit, often resulting in an initial burst release that is beneficial for early neuroprotection [86]. Alternatively, internal luminal fillings, such as hydrogels or microparticles loaded with tacrolimus, can be placed inside the conduit to provide a more controlled and prolonged release during the critical phases of nerve regeneration [39,40].

### 5.2. Nerve Wraps and Sleeves

Nerve wraps and sleeves are designed to enclose the nerve repair site, including suture lines or crush injuries. Their primary functions are to prevent neuroma formation, reduce fibrous scarring, and protect the regenerating nerve from the surrounding tissue environment [87,88]. These systems can be loaded with therapeutic agents to provide localized immunosuppressive and neuroprotective effects directly at the site of injury [19].

Biodegradable films composed of polymers such as PGA, PLGA, or silk fibroin are engineered to gradually degrade and release drugs in a sustained manner. These films can also be fashioned into tubular wraps or sleeves, creating an advanced delivery system [89]. Alternatively, natural membranes, such as human amniotic membrane, show promise as wraps due to their anti-inflammatory properties and ability to promote regeneration [90]. Recent advances have refined these systems to optimize localized drug delivery and therapeutic efficacy. Bilayer and multilayer designs act as drug reservoirs, enabling an initial high-dose release followed by sustained delivery. This is an ideal pharmacokinetic profile for supporting nerve healing and functional recovery [91].

### 5.3. Injectable Hydrogels

Injectable hydrogels offer a minimally invasive approach for local drug delivery. These systems are particularly advantageous for filling irregularly shaped nerve defects or for direct application around repair sites [92]. Hydrogels can conform to the anatomy of the injury, serving as both structural scaffolds and localized tacrolimus delivery platforms. Both natural hydrogels (including collagen, fibrin, hyaluronic acid, and chitosan), and synthetic polymers (such as polyethylene glycol (PEG)) offer versatile options for localized delivery. Their injectability and in situ gelation enable precise, uniform application to complex nerve injury sites. Key properties such as tunable release kinetics and tunable bioactivity achieved through the control of matrix stiffness, degradation rate, and the incorporation of bioactive cues such as adhesion motifs or growth factor-binding domains enhance regenerative potential by allowing sustained drug delivery over days to weeks while simultaneously supporting cellular integration and axonal growth [34,93,94]. Encapsulation strategies range from direct dissolution of the drug to the incorporation of drug-loaded micro- or nanoparticles, enabling multiphase release profiles tailored to the therapeutic needs of nerve repair [95].

### 5.4. Other Scaffolds and Advanced Delivery Platforms

Scaffold-mediated LDSs serve a dual role in nerve repair by providing structural support for regenerating axons while enabling localized, sustained release of therapeutic agents [96]. Engineered from biocompatible polymers, ceramics, or composite materials, these scaffolds are typically designed with a porous architecture that facilitates cellular infiltration, supports Schwann cell migration [97], and promotes integration with host nerve tissue [98]. Among these, fibrous scaffolds, particularly aligned electrospun fibers, have demonstrated strong potential in guiding axonal regrowth due to their anisotropic structure [99]. When loaded with therapeutic agents, these scaffolds establish a neuroregenerative microenvironment that combines mechanical support with sustained immunomodulatory and pro-regenerative signaling [99]. An additional strategy involves cell-laden scaffolds, in which vascular endothelial growth factor A (VEGF-A) is co-delivered with therapeutic cells such as Schwann cells or stem cells. This approach enhances cell survival and function while amplifying neurotrophic factor secretion, fostering a synergistic regenerative response at the injury site [100]. A summary of various prominent LDS strategies for nerve repair is provided in Table 1. Although many of the systems described utilize alternative therapeutic agents, the underlying delivery strategies are directly translatable to tacrolimus.

In parallel, advanced drug delivery systems, including nanocarriers such as liposomes and polymeric nanoparticles, are being explored for their ability to deliver therapeutics directly to target cells, enabling on-demand release while limiting systemic exposure [123]. Hu et al. developed a unique scaffold combining reduced graphene oxide, a GelMA hydrogel, and brain-derived neurotrophic factor (BDNF) on butterfly wing structures. This scaffold improved neuronal growth and alignment due to its electrical conductivity and surface structure. When incorporated into NGCs, it demonstrated efficacy in 10 mm sciatic nerve defects in rats. Muscle recovery in treated animals was comparable to that achieved with autografts, underscoring the scaffold’s robust regenerative effect [124].

In another study, researchers developed a nanoparticle system by encapsulating a stromal cell-derived factor-1α (SDF-1α) mimetic peptide within a metal–organic framework (ZIF-8). These SDFP@ZIF-8 nanoparticles were embedded within a porous gelatin hydrogel (S@Z@G nanogel), creating a biocompatible and mechanically stable delivery platform. These nanoparticles influenced multiple cell types critical for nerve repair, including macrophages, endothelial cells, and Schwann cells. Mechanistically, they promoted the phenotypic transition of pro-inflammatory M1 macrophages into pro-healing M2 macrophages by inhibiting the HIF-1 pathway and glycolysis, thereby reducing inflammation. When applied to injured rat nerves, this nanogel promoted nerve regeneration by supporting angiogenesis, remyelination, and overall functional recovery [125]. This approach exemplifies how modifying the local injury environment can improve nerve healing. Collectively, these advanced delivery platforms combine structural guidance with biochemical support, offering substantial potential to synergistically enhance nerve regeneration and functional recovery in clinical settings.

## 6. Preclinical Evidence of Local Tacrolimus Delivery in a Nerve Injury Model

Building onto these mechanistic insights, preclinical studies have directly evaluated local tacrolimus delivery in nerve injury models. In vivo mouse and rat models provide essential platforms to assess the biocompatibility, functionality, and safety of therapeutic strategies, supporting translation to clinical trials. Tacrolimus has been incorporated into diverse delivery systems, including synthetic base polymers, bio-derived materials, inorganic matrices, and composite constructs, to enable targeted drug release at the site of neural injury [16]. Our previous work demonstrated that local tacrolimus delivery notably enhanced motor and sensory nerve regeneration in fresh nerve allografts, yielding outcomes comparable to systemic immunosuppression or nerve isografts. Selection of a delivery platform is guided by sustained release, local retention, and biocompatibility, aiming to maximize neuroregenerative potential while minimizing systemic toxicity [74]. While this review focuses on peripheral nerve regeneration, the inclusion of related models provides mechanistic insights into tacrolimus-mediated neuroregeneration across neural tissues. Table 2 summarizes key preclinical studies of tacrolimus. Table 2 focuses on peripheral nerve injury and repair models, and highlights related applications in corneal, graft, spinal cord, and transplant models.

Although substantial progress has been made, static tacrolimus delivery systems face several limitations such as short drug duration, lack of spatial or temporal control, and potential toxicity. To address these challenges, emerging stimuli-responsive platforms offer precise, adaptable control over therapeutic release. For instance, a thermoresponsive nanogel incorporating bupivacaine and photothermal hollow gold nanoparticles enabled repeated near-infrared (NIR) light-triggered drug release, achieving pulsatile and prolonged anesthetic effects at the sciatic nerve site in vivo, without inducing local toxicity. This system illustrates that optical stimulation can be harnessed for non-invasive, spatiotemporal control of drug release [140]. Similarly, a responsive cascade drug delivery scaffold (RCDDS) was developed to sequentially release vitamin B12 and NGF for early inflammation suppression and subsequent neuroregeneration. The system also permitted the ultrasound-triggered modulation of drug release kinetics, enabling real-time adjustment according to patient-specific therapeutic requirements. In rat sciatic nerve injury models, RCDDS facilitated functional recovery comparable to autografts [141]. Another approach employed an electrically responsive nanocomposite of graphene oxide (GO) within a poly(3,4-ethylenedioxythiophene) (PEDOT) matrix, enabling voltage-controlled release of the neurotrophic agent 7,8-dihydroxyflavone (7,8-DHF). The drug was π–π stacked onto GO and co-deposited in the PEDOT matrix, while a dopamine-grafted chitosan coating improved biocompatibility [142]. Upon electrical stimulation, dose-dependent release of 7,8-DHF promoted dorsal root ganglion neurite outgrowth, Schwann cell migration, synaptic function, and mitochondrial biogenesis, demonstrating the system’s potential to integrate multiple regenerative signals under user control [143].

**Figure 2:**
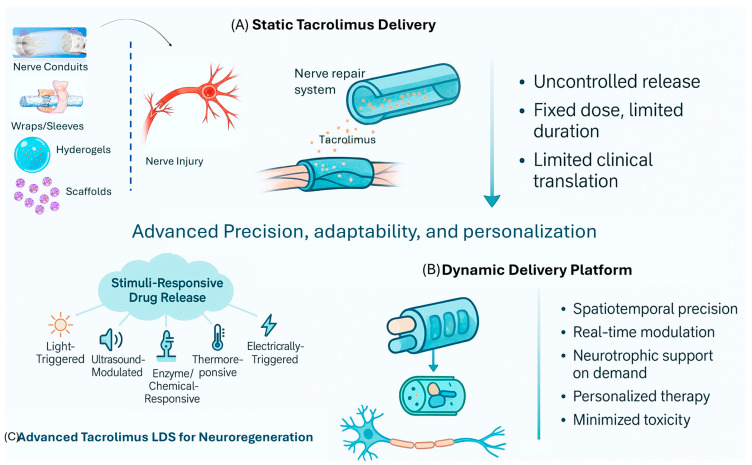
Existing and emerging local delivery systems for tacrolimus that can be used during surgical nerve repair. Created in BioRender. Katturajan, R. (2026) https://BioRender.com/shaakmn, accessed on 27 April 2026.

The method of drug incorporation critically governs tacrolimus release kinetics by modulating polymer degradation rates and drug distribution within the matrix. This directly determines local tacrolimus concentrations achieved at the nerve injury site, which in turn dictate biological outcomes. Evidence shows that optimal local tacrolimus concentrations (~1.79 ± 0.01 ng/mL) significantly enhance Schwann cell proliferation and axonal regeneration via the FKBP-52/MAP kinase/ERK pathway, independent of its immunosuppressive calcineurin inhibition [144,145]. Controlled local delivery systems, such as fibrin gels and electrospun polymer wraps, achieve sustained, degradation-matched tacrolimus release, resulting in accelerated axonal regeneration, enhanced myelination (upregulated MBP expression), and improved functional recovery without systemic toxicity [54,146]. Conversely, burst release from poorly optimized incorporation methods leads to supra-optimal concentrations that fail to provide additional benefits or cause local irritation [144,147]. Therefore, an integrative discussion linking material degradation profiles, achieved local tacrolimus concentrations, and regenerative outcomes (axonal regeneration rate, myelination, functional recovery) is essential when reporting nerve regeneration studies involving tacrolimus-eluting implants. Collectively, these responsive delivery systems illustrate compelling strategies to overcome the limitations of systemic exposure to tacrolimus. However, each maintains its own set of risks and uncertainties. In their simplest form, static tacrolimus delivery systems, (nerve conduits, wraps, sleeves, hydrogels, and scaffolds) provide localized drug exposure but are limited by uncontrolled release kinetics, fixed dosing, and short therapeutic duration. Additionally, several materials described in Table 2, such as non-degradable inorganic nanoparticles, metallic components, and complex multi-phase composites, raise concerns related to long-term tissue residency, migration, and regulatory approval for neural or ocular applications [148]. Dynamic, stimuli-responsive delivery platforms offer enhanced spatiotemporal control and on-demand neurotrophic support. However, their clinical translation remains constrained by material complexity, external activation requirements, batch-to-batch variability, and challenges in scalable GMP-compliant manufacturing. As a whole, these limitations serve to further highlight the need for biodegradable and regulatory-compliant material designs that are effective yet readily translated clinically (Figure 2).

## 7. Clinical Studies and Research Opportunities

Preclinical studies demonstrate the promising neuroregenerative effects of local tacrolimus delivery, however, clinical applications remain largely confined to systemic administration, primarily for immunosuppression in nerve allografts [149]. Systemic tacrolimus carries a black box warning from the FDA regarding serious adverse effects as discussed previously, as well as an elevated risk of malignancies. Though tacrolimus (Prograf) has been approved for kidney, heart, liver, lung, and other organ transplants, at this time, no neuroregenerative indications are FDA-approved.

To date, clinical research evaluating localized tacrolimus delivery for peripheral nerve repair remains extremely limited. A particularly illustrative example is the clinical trial NCT00950391, led by Dr. Thomas Tung at Washington University in St. Louis. This prospective study was designed to assess functional recovery following peripheral nerve injury in patients treated with systemic tacrolimus at a dose of 3 mg/day, targeting blood concentrations of 3–6 ng/mL for up to one year. Initiated in 2010, the trial aimed to translate preclinical evidence of tacrolimus-mediated neuroregeneration into clinical outcomes. However, despite its strong scientific rationale, the study was withdrawn in 2018 before completion due to funding constraints, leaving a significant gap in clinical data regarding the therapeutic potential of tacrolimus in nerve repair. Barriers to clinical translation include safety concerns related to systemic exposure, lack of approved localized delivery devices, and the complexities of designing trials capable of isolating neuroregenerative effects from immunosuppressive activity. Other clinical studies have investigated nerve repair systems utilizing natural and synthetic biomaterials, including plant-based polymers such as scaffolds or drug delivery platforms (summarized in Table 3). Innovations such as the polymeric matrix devices described by Washington et al. (US11529318B2) provide local, sustained tacrolimus release directly to injured nervous tissue, representing important steps toward clinical translation [150]. Similarly, our group also introduced fibrin and polymer-based FK506 delivery systems that enable localized, sustained tacrolimus release at nerve repair sites, achieving therapeutic levels (>5 µg/mL/day for ≥14 days) while minimizing systemic exposure [151]. While several nerve repair systems have entered clinical evaluation, most studies reveal limitations in long-term efficacy, scalable manufacturing, or regulatory readiness, underscoring the need for next-generation delivery platforms.

Ample opportunities exist to address current limitations and optimize the therapeutic potential of tacrolimus in nerve regeneration. One priority is the development of biocompatible, mechanically suitable local delivery systems that enable sustained and controlled drug release (as described in Section 5 and Section 6). Another promising avenue is combination therapies that integrate tacrolimus with neurotrophic factors, extracellular vesicles, or immunomodulators to synergistically enhance regenerative outcomes. Additionally, patient-specific, stimuli-responsive delivery platforms capable of adapting to the dynamic post-injury microenvironment may further enable precision medicine in nerve repair. Rigorous safety and efficacy trials, alongside scalable and reproducible manufacturing processes, will be essential to clarify the risk–benefit profiles and facilitate widespread adoption. Achieving these goals will require ongoing interdisciplinary collaboration spanning biomaterials science, pharmacology, and clinical research to successfully translate localized tacrolimus delivery from the bench to bedside.

A clinically viable tacrolimus delivery system for peripheral nerve repair should provide sustained and reproducible local drug release within a therapeutic window, minimize systemic exposure, remain biocompatible, and be manufacturable under quality-system requirements. For combination products, lead-center assignment depends on the primary mode of action (PMOA), and the regulatory pathway may vary by product type. Biomaterials with prior device precedent, such as collagen-based or synthetic biodegradable polyester-based nerve wraps and other biodegradable nerve-guidance platforms, may be more readily translatable than highly complex multi-component systems, although the final regulatory route depends on the full product design and the added drug constituent. Biocompatibility evaluation should follow the FDA’s risk-based ISO 10993 framework, and early IND/IDE planning is advisable for clinical studies [157].

## 8. Regulatory and Manufacturing Challenges in Tacrolimus Delivery Systems

Despite encouraging preclinical and early clinical findings, the translation of tacrolimus delivery systems faces substantial regulatory and manufacturing challenges. Many current platforms incorporate materials or device architectures that lack precedent in FDA-approved neural or ophthalmic implants, complicating safety assessment and approval pathways. Combination products integrating drugs, biomaterials, and external activation modalities further increase regulatory complexity.

From a manufacturing perspective, scalable production remains limited by complex synthesis protocols, poor batch reproducibility, and challenges in sterilization and long-term storage stability. These limitations have contributed to the stagnation of several promising delivery systems at the preclinical stage, underscoring the need for simplified, biodegradable, and GMP-compatible designs.

## 9. Conclusions

Peripheral nerve injuries (PNIs) pose a substantial challenge, highlighting the urgent clinical need for advanced therapeutic strategies. Localized tacrolimus delivery systems offer a promising approach by combining the drug’s dual capacity for immunomodulation and direct neuroregeneration, providing targeted, site-specific administration that minimizes systemic toxicity. Preclinical studies demonstrate that platforms ranging from nerve conduits and wraps to injectable hydrogels and nanocarriers effectively deliver tacrolimus, offering structural support, controlled release profiles, and an optimized microenvironment for axonal regeneration and remyelination. Looking forward, the integration of patient-specific and stimuli-responsive technologies holds immense potential to further refine delivery precision and therapeutic efficacy. Successful clinical translation will therefore require coordinated efforts to simplify material composition, align device design with established regulatory frameworks, establish scalable GMP-compliant manufacturing processes, and validate long-term safety and efficacy in clinically relevant models. By leveraging the neuroregenerative potential of tacrolimus through these advanced localized delivery systems, the field is poised to redefine the standard of care in nerve repair.

## Figures and Tables

**Table 1:** Local delivery system for repairing nerves.

Form	Material(s)	Therapeutic Agent(s)	Delivery Mechanism	Reported Results	Ref.
Nerve Guidance Conduits	Chitosan, HNTs, epichlorohydrin	4-AP	Physical axon guidance; sustained drug release	Autograft-equivalent repair, functional recovery, myelin formation	[101]
	PLGA	NGF	Concentric tube; controlled local and sustained release	Significant myelination, muscle innervation, and bioactive NGF for 28 days	[102]
	Chitosan and collagen	Schwann cells	Encapsulated cells providing regenerative support	Directed axon extension	[103]
	PBS/PLA/fibroin-agarose (sandwich structure)	Ciprofloxacin	Porous inner/outer layers for controlled drug release	Anti-infection effect and guided nerve regeneration	[104]
	PLA and polypropylene yarn	Schwann cells	Multichannel structure optimizing cell adhesion and proliferation	Optimized Schwann cell adhesion and proliferation	[105]
	PLCL, gelatin hydrogel	NGF	Non-collapsible scaffold structure with local NGF release	Effective axonal regeneration, remyelination, and enhanced recovery	[106]
Nerve Wraps	PLA/PCL co-polymer	Ibuprofen	Anti-inflammatory wrap with drug loading	Improved axonal growth, sensory recovery, and upregulation of neurotrophic factors	[107]
	mPEG-PLGA	Rapamycin	Early controlled release; physical barrier	Enhanced regeneration, reduced adhesions, improved myelination and recovery	[108]
	EVA/PLGA co-polymer	Ibuprofen sodium, Sulindac sulfide	Localized controlled release at injury site	Increased neurite growth, improved functional recovery	[109]
	N3-GelMA, DBCO-GelMA, MPEG-PCL (3D printed bandage)	XMU-MP-1 (nanodrug)	Self-adhesive bandage; nanodrug-releasing grating layer	Enhanced Schwann cell proliferation and migration; promoted regeneration	[110]
Nerve Sleeves	Parylene C/gold/PEDOT: PSS/silicone/polyimide	–	Microelectrode cuffs for stimulation and recording	High-resolution nerve interfacing; fascicle-specific recording and stimulation	[111]
	Chitosan conduit + terpolymer rods	17-β-estradiol (E2)	Sustained local release	Increased axon numbers, reduced inflammation	[112]
	PLGA microspheres + chitosan conduit	NGF + bFGF	Microspheres within the conduit enabling synergistic release	Significantly improved conduction, histology, and functional outcome	[113]
Hydrogels	PNIPAm/alginate/tannic acid	Rhein	Self-curling hydrogel with microneedles and electrical stimulation	Enhanced repair, reduced inflammation, improved function	[114]
	4-arm PEG-NHNH2, o-Dex, neural stem cells	Cetuximab + FTY720	Hydrogel delivering NSCs and drugs; promotes differentiation and inhibits glial scarring	Axonal regeneration, inhibited scar formation, and optimal recovery	[115]
	GelMA + QK-nanoliposomes	QK peptide (VEGF mimic)	Injectable hydrogel for sustained release; promotes revascularization	Enhanced axon regeneration, M2 macrophage polarization, improved recovery	[116]
	GelMA	bFGF	Porous microspheres for sustained bFGF delivery	Improved NSC proliferation, differentiation, nerve regeneration	[117]
Scaffolds	CNTs@GelMA/PLLA composite	–	Aligned conduit with conductive GelMA/CNTs hydrogel, enabling endogenous stimulation	Enhanced Schwann and DRG growth, myelination, axonal outgrowth, and functional recovery	[118]
	P(MMD-co-LA) nanofibers	Deferoxamine (DFO)	Aligned nanofibers guiding regeneration, DFO release promoting angiogenesis	Reduced inflammation, improved vascularization, and recovery	[119]
	Chitosan/silk fibers + SKP-SC ECM	Pre-differentiated Schwann cells	Scaffold with cell-secreted ECM provides a pro-regenerative environment	Comparable to autografts in promoting myelination and reinnervation	[120]
	RCP/pJUN-PSPF@PGA scaffold	c-Jun plasmids	RGD-tagged scaffold delivering plasmids to Schwann cells	Sustained NGF/BDNF/VEGF expression; regeneration comparable to autografts	[121]
	PCL/CNT-PDA fibrous scaffold	BDNF	Aligned topography and conductive cues; PDA coating enables sustained BDNF release	Promoted Schwann cell growth and myelination genes; in vivo regeneration like autografts	[122]

Notes: HNT: Halloysite Nanotube; 4-AP: 4-Aminopyridine; PLGA: Poly(lactic-co-glycolic acid); NGF: Nerve Growth Factor; PBS: Poly-butylene succinate; MPEG: Methoxy Polyethylene Glycol; EVA: Ethylene Vinyl Acetate; bFGF: Basic Fibroblast Growth Factor; PLA: Polylactic Acid; PCL: Polycaprolactone; PEG: Polyethylene Glycol; DBCO-GelMA: Dibenzocyclooctyne-Functionalized Gelatin Methacrylate; PLCL: Poly(lactide-co-caprolactone); CNT: Carbon Nanotube; GelMA: Gelatin Methacrylate; QK: VEGF-Mimetic Peptide; NSC: Neural Stem Cell; RCP: Recombinant Collagen Peptide; PDA: Polydopamine; BDNF: Brain-Derived Neurotrophic Factor; ECM: Extracellular Matrix; SKP-SC: Skin Precursor-Derived Schwann Cell; P(MMD-co-LA): Poly(mannitol dicarboxylate-co-lactic acid); DFO: Deferoxamine.

**Table 2:** Preclinical studies of tacrolimus-LDSs demonstrating enhanced therapeutic efficacy in nerve regeneration.

Form	Materials/Composites	Fabrication Technology	Key Findings	Target Nerve Type	Condition/Model	Release Profile	Ref.
Nerve guidance conduit (Tac-eluting)	PLCL	Extrusion, diffusion hole drilling, assembly	Improved neurite outgrowth, thermal sensitivity, locomotion, and sensory regeneration	Sciatic nerve	Peripheral nerve injury/Rats	Sustained (~12.2 ng/day)	[126]
Nerve conduit with aligned fibers	PCL + Tacrolimus	Electrospinning (aligned/random fibers)	Aligned fibers guide neurite outgrowth	Peripheral nerve	Peripheral nerve injury/Rats	Sustained over several weeks	[127]
Tac-loaded fibrin/PLGA conduit	Fibrin hydrogel, PLGA microspheres, Tacrolimus	Single emulsion solvent evaporation	100 ng/mL released for 35 days, stem cell viability for 7 days	Peripheral nerve	Peripheral nerve injury/Rats	Sustained (high dose), rapid (low dose)	[128]
ADSC–Matrigel injectable system	Rat ADSCs, Matrigel, Tacrolimus	Cell pretreatment, mixing, injection	Enhanced nerve regeneration in rats	Peripheral nerve	Nerve crush injury/Rats	Not applicable	[75]
Core–shell nerve wrap	PCU + Tacrolimus	Co-axial electrospinning	Accelerated regeneration, improved recovery	Peripheral nerve	Surgical nerve repair/Rats	Biphasic, sustained >31 days	[54]
Biodegradable nanofiber for eye nerves	PCU + Tacrolimus	Electrospinning	Limbal innervation, sensory axon growth	Corneal nerves	Neurotrophic keratopathy/Rats	Topical release ~4 weeks	[39]
Polyester urethane urea wrap	Tacrolimus-impregnated PEUU	Electrospinning	Improved myelination, faster recovery	Infraorbital nerve	Infra-orbital nerve transection/Rats	Sustained ~6 weeks	[6]
Coaxial electrospun fiber sheets	PCL + Tacrolimus	Coaxial electrospinning	Reduced immune response to allografts	Sciatic nerve	Nerve allograft/Rats	Controlled over several weeks	[129]
Collagen membrane system	Collagen + Tacrolimus + fibrin glue	Soaking and application	Promoted regeneration after traction injury	Sciatic nerve	Traction injury/Rats	Sustained ~4 weeks	[130]
Mixed thermosensitive hydrogel	Poloxamer + PLX + Tacrolimus	Copolymer synthesis	Improved regeneration, reduced systemic side effects	Sciatic nerve	Traction injury/Mice	Sustained ~1 month	[131]
In situ thermosensitive hydrogel	Tacrolimus, P-Lys-Ala-PLX, Poloxamer	In-situ gelling formulation	Improved allograft survival, no systemic toxicity	Skin allograft	Tail skin transplant/Rats	Sustained >90 days	[132]
Maghemite nanospheres	Tacrolimus, maghemite, PEG	Hydrothermal + co-precipitation	Improved recovery from spinal cord injury over free drug	Spinal cord	Spinal cord injury/Rats	Time-dependent, pH-sensitive	[133]
Collagen hydrogel	Collagen + Tacrolimus	Mixing + gelatinization	Enhanced neurogenesis without systemic effects	Spinal cord	Spinal cord injury/Rats	Sustained ~28 days	[134]
Electrospun nanofiber conduit	Tacrolimus + PLGA	Electrospinning	Long-gap repair, restored nerve/muscle function	Sciatic nerve	Peripheral nerve injury/Rats	Sustained release	[127]
Polymeric micelles	Tacrolimus + PCL-PEO block copolymer	Dissolution + dialysis	Enhanced sciatic nerve recovery	Sciatic nerve	Crushed nerve/Rats	Sustained release	[135]
Exosome-based delivery	Tacrolimus, ADSC-derived exosomes	Exosome isolation	Suppressed macrophage autophagy, improved regeneration	Sciatic nerve	Crush injury/Mice	Local delivery	[136]
Tacrolimus-eluting disk	Tac-loaded PCL	Solvent casting	Allograft survival >200 days	Vascularized composite allograft	Transplant model/Mice	Controlled intra-graft release	[137]
Elastomeric polymer matrix	PEUU + Tacrolimus	Electrospinning	Stimulated axon growth in retinal ganglion cells	Optic nerve	Acute injury/Mice	Sustained release	[138]
PLGA/PLA double-walled microspheres	PLGA, PLLA + Tacrolimus	Oil-in-oil emulsion	>180-day allograft survival	Hindlimb transplant	Orthotopic transplant/Rats	Sustained ~146 days	[139]

Notes: PLGA: Poly(lactic-co-glycolic acid); PCL: Polycaprolactone; PEG: Polyethylene Glycol; ADSC: Adipose-Derived Stem Cell; ECM: Extracellular Matrix; RGC: Retinal Ganglion Cell; SFI: Sciatic Functional Index; CNS: Central Nervous System; PEO: Polyethylene Oxide; Micelles: Nano-scale colloidal carriers for drug delivery; NSC: Neural Stem Cell; PLX: Pluronic F-127; P-Lys-Ala: Poly(L-alanine-lysine); PCU: Polycarbonate Urethane; PEUU: Poly(ester urethane) Urea; PLCL: Poly(L-lactide-co-caprolactone); TAC: Tacrolimus; PEUU: Polyester urethane urea.

**Table 3:** Clinical studies on nerve repair systems and devices.

Nerve Repair System	Material	Mechanism	Conditions and Indication-Specific Limitations	No. of Subjects	Trial ID/Reference
Polynerve Nerve Conduit	Co-polymer of PCL and PLLA	Biodegradable tubular scaffold with micro-grooved internal architecture guiding regeneration	Evaluated for short-gap wrist and hand injuries; designed for structural guidance rather than biologic augmentation or controlled drug delivery; long-term functional outcomes remain limited	17	NCT02970864
Avance^®^ Nerve Graft	Decellularized peripheral nerve	Natural extracellular matrix scaffold supporting regeneration across nerve gaps	FDA-approved for peripheral nerve repair; efficacy decreases with increasing gap length and delayed repair; platform does not support sustained local drug delivery or modulation of the regenerative microenvironment	5000	NCT01526681
Nerve Wrap and Conduit	Fibrin	Provides biological support and stabilizes repair site, promoting regeneration	Primarily adjunctive; limited mechanical durability and rapid degradation; not intended for long-gap repair or active acceleration of axonal regeneration	37	NCT01573650
Biodegradable Conduit Small-Gap Tubulization	Degradable biomaterial (not specified)	Bridges small nerve gaps	Limited to acute, small-gap injuries; complicating reproducibility and mechanistic interpretation; does not address delayed regeneration	150	NCT03359330
SilkBridge Conduit	Silk fibroin-based scaffold	Biomimetic scaffold recruiting host cells to regenerate nerve tissue	Early-stage clinical evaluation with very small cohort; approved indication limited to short gaps; not designed for pharmacologic loading or controlled release	4	NCT03673449
Neuromaix Biodegradable Conduit	Porcine collagen	Structural and guidance scaffold for nerve regeneration	Clinically evaluated primarily in biopsy or sensory applications; approved for structural repair but not biologic augmentation; lacks capacity for sustained therapeutic delivery	20	NCT01884376
Bionic Nerve Scaffold Polymer Conduit	Collagen	Biomimetic collagen scaffold with microchannel architecture guiding nerve growth	Limited to short sensory nerve gaps; complex architecture increases manufacturing demands; not evaluated for long-gap repair or accelerated regeneration	10	NCT03780855
Reaxon^®^ Nerve Guide	Chitosan	Bridges nerve defects up to 26 mm	FDA-cleared for short-to-moderate gaps; functional recovery remains length-dependent; platform does not incorporate active neurotrophic or drug-delivery mechanisms	46	NCT02459015
Nerve Tube	Chitosan	Applied over microsurgical repair to prevent scar formation	Protective rather than regenerative; does not promote axonal elongation or address denervation-associated muscle atrophy	100	NCT02372669
Hydrogel-Facilitated Nerve Repair	Polyethylene Glycol	Promotes axon fusion, restores compound action potentials, and may prevent Wallerian degeneration	Indication limited to acute injuries; long-term functional outcomes and generalizability remain under evaluation; not designed for sustained therapeutic release	18	NCT02359825
Nerve Wrap	Collagen	Surrounds repaired nerve to reduce scarring and neuropathic pain	FDA-cleared as an adjunctive barrier; does not directly enhance axonal regeneration or reinnervation kinetics	52	[152]
Neurolac^®^ Nerve Guide	PLCL	Synthetic biodegradable conduit guiding axonal regrowth	Approved for nerve guidance; mechanical mismatch with native nerve and foreign-body response reported; declining use highlights performance limitations	34	[153]
VersaWrap^®^ Nerve Protector	Plant-based hydrogel matrix (unspecified)	Acts as a barrier to epineural scarring and adhesions; wraps nerve after neurolysis	FDA-cleared for nerve protection; functions as a passive barrier and does not support regeneration or pharmacologic modulation	20	[154]
Nerve Wrap	Porcine extracellular matrix	Scaffold and barrier reducing scarring, supports healing	Approved for scar reduction; xenogeneic origin limits integration of controlled drug delivery or bioactive signaling	102	[155]
Bio 3D Nerve Conduit	Dermal fibroblast	Scaffold using patient-derived cells printed into a nerve guide	Proof-of-concept clinical use; highly personalized manufacturing presents scalability and regulatory challenges; not yet adaptable for combination drug–device strategies	3	[156]

Notes: ECM: Extracellular Matrix; PEG: Polyethylene Glycol; PCL: Polycaprolactone; PLA: Polylactic Acid; PLGA: Poly(lactic-co-glycolic acid); Chitosan: Biopolymer derived from chitin; BDNF: Brain-Derived Neurotrophic Factor; VEGF: Vascular Endothelial Growth Factor; NCT: ClinicalTrials.gov identifier; RCT: Randomized Controlled Trial; Sural nerve: A sensory nerve commonly used for biopsy in clinical studies; PCL: Poly ε-caprolactone; PLLA: Poly(L-lactic acid); PLCL: Poly(L-lactide-co-ε-caprolactone).

## Data Availability

No new data were created or analyzed in this study. Data sharing is not applicable to this article.
